# Supplementary material for: A blood-based biomarker workflow for optimal tau-PET referral in memory clinic settings
Source: Nat Commun. 2024 Mar 14;15:2311. doi: 10.1038/s41467-024-46603-2 (PMC10940585; doi:10.1038/s41467-024-46603-2)
Supplement: Supplementary file 1 — Supplementary Information [file 41467_2024_46603_MOESM1_ESM.pdf]

Supplementary Information:

A blood-based biomarker workflow for optimal tau-PET referral in memory clinic settings

Supplementary Figure 1. Combining age and *APOE* ε4 status to individual biomarkers did not lead to increases in saved scans.

Supplementary Figure 2. Defining tau-PET positivity with a validated visual read method leads to similar results in terms of avoided scans.

Supplementary Figure 3. Defining tau-PET positivity with a validated visual read method leads to similar results in terms of positive predictive values.

Supplementary Figure 4. Tau-PET scans avoided and screening accuracy in SCD-MCI individuals in BioFINDER-2 and TRIAD.

Supplementary Figure 5. Tau-PET scans avoided and screening accuracy in all-cause dementia individuals in BioFINDER-2 and TRIAD.

Supplementary Figure 6. Distribution of tau-PET visual read results by plasma p-tau217 status in BioFINDER-2 participants.

Supplementary Figure 7. Raw cognitive trajectories of prognostic analyses based on the plasma p-tau217 referral cutoff.

Supplementary Table 1. Negative and positive predictive values for each biomarker and screening strategy in BioFINDER-2.

Supplementary Table 2. Negative and positive predictive values for each biomarker and screening strategy in TRIAD.

Supplementary Table 3. Negative and positive predictive values for tau-PET positivity defined with visual read in BioFINDER-2.

Supplementary Figure 1. Combining age and *APOE* ε4 status to individual biomarkers did not lead to increases in saved scans.

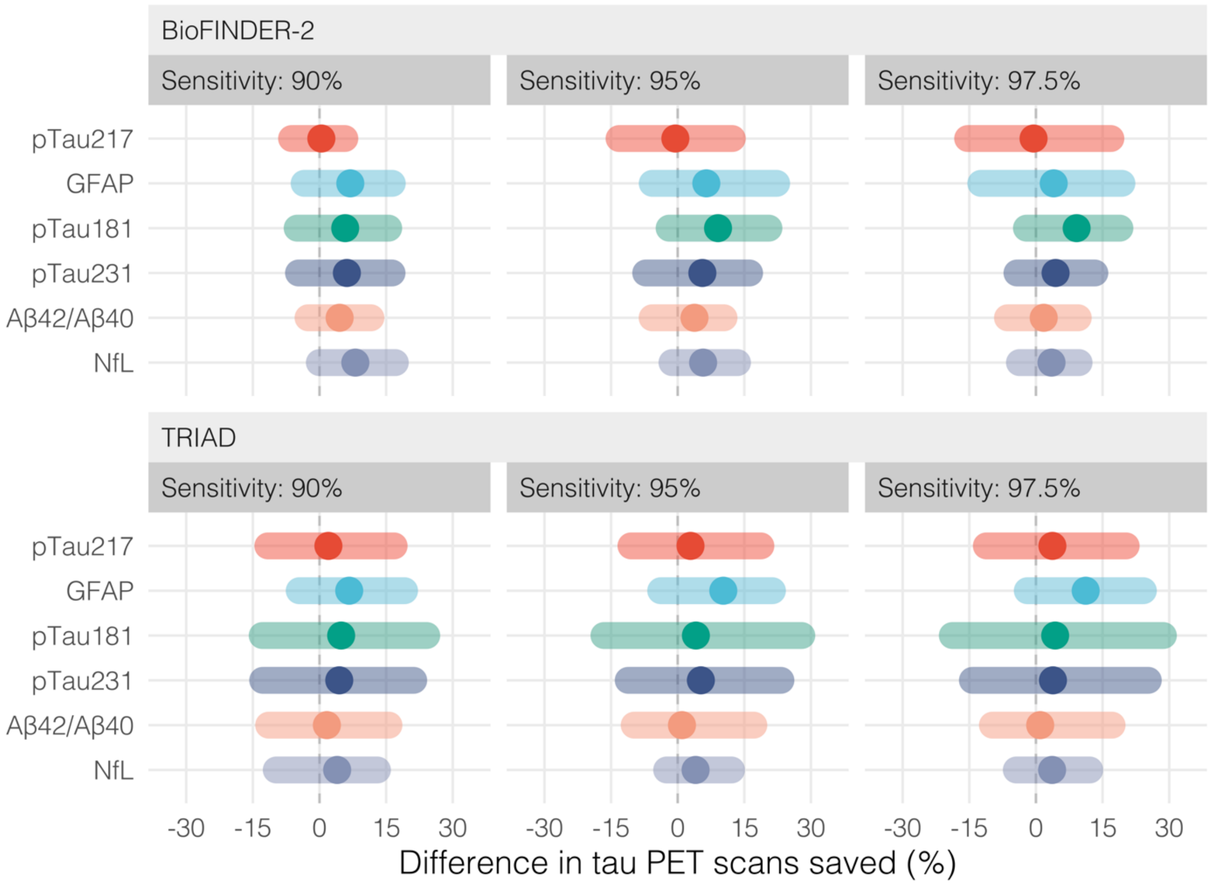

These forest plots indicate the percentage point difference in the proportion of avoided scans when comparing the effect of combining age and *APOE* ε4 status to each biomarker versus each individual biomarker without these covariates. Biomarkers are on the y-axis, and the x-axis corresponds to the difference in saved scans in the model with covariate compared with each individual biomarker alone. Results are shown on the top row for BioFINDER-2 (n=548) and bottom row for TRIAD (n=179). Each of the panels corresponds to this difference when evaluating a certain sensitivity strategy for the screening cutoffs. The 95% confidence intervals were derived with bootstrapping (n=100). The dashed line corresponds to no difference in saved scands by adding these covariates. Source data are provided as a Source Data file. PET = positron emission tomography; Aβ = amyloid-beta; pTau = phosphorylated tau; NfL = neurofilament light; GFAP = glial fibrillary acidic protein; APOE = apolipoprotein E.

Supplementary Figure 2. Defining tau-PET positivity with a validated visual read method leads to similar results in terms of avoided scans.

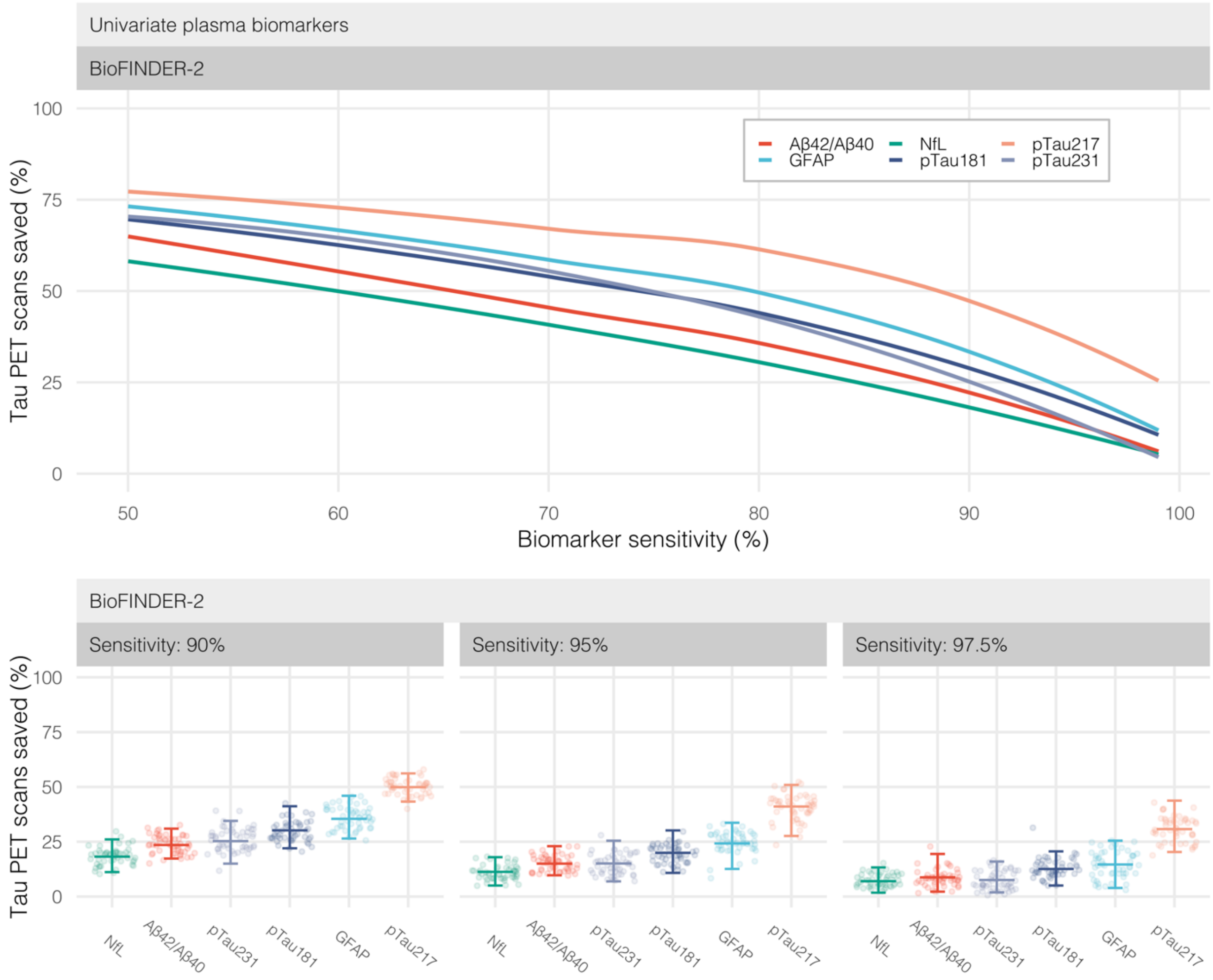

This figure shows the percentage of tau-PET scans saved when screening BioFINDER-2 patients for tau-PET-positivity, as defined by a validated visual read method (n=539), with continues sensitivity values in the top row, and with screening strategies varying at sensitivity values (90%, 95%, 97.5%) in the bottom row. Source data are provided as a Source Data file. PET = positron emission tomography; Aβ = amyloid-beta; pTau = phosphorylated tau; NfL = neurofilament light; GFAP = glial fibrillary acidic protein.

Supplementary Figure 3. Defining tau-PET positivity with a validated visual read method leads to similar results in terms of positive predictive values.

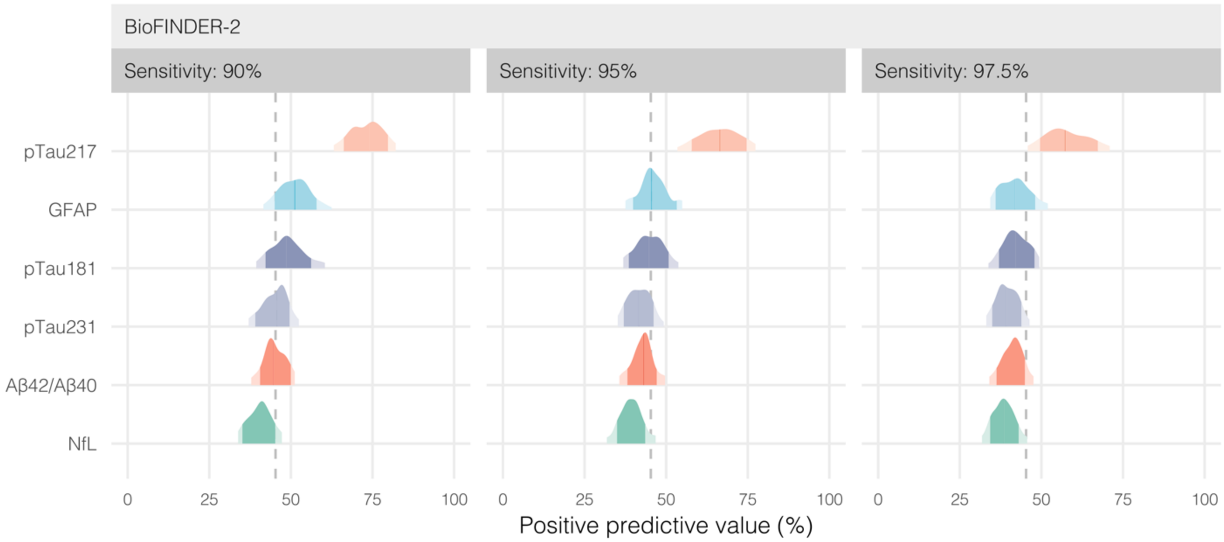

This figure shows the percentage of tau-PET positive individuals within the group theoretically refered to a tau-PET scan with each biomarker, i.e. the positive predictive value (PPV). This is shown for BioFINDER-2 patients, with tau-PET positivity defined with a validated visual read method (n=539). The PPVs are shown for different screening strategies varying at sensitivity values (90%, 95%, 97.5%), and the dashed line represents the prevalence of tau-PET positivity defined with the visual read method (47%). Source data are provided as a Source Data file. PET = positron emission tomography; Aβ = amyloid-beta; pTau = phosphorylated tau; NfL = neurofilament light; GFAP = glial fibrillary acidic protein.

Supplementary Figure 4. Tau-PET scans avoided and screening accuracy in SCD-MCI individuals in BioFINDER-2 and TRIAD.

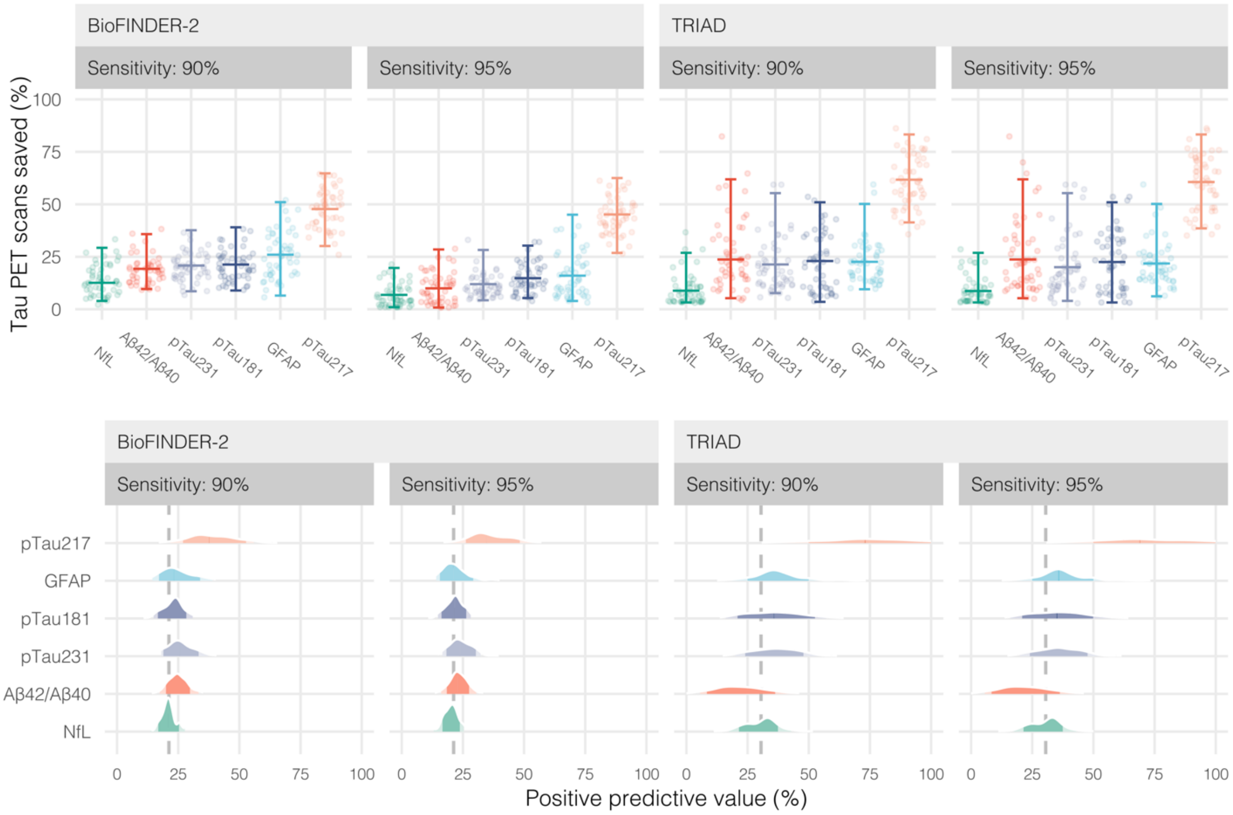

The top panels show the percentage of tau-PET scans saved when screening SCD-MCI individuals for tau-PET-positivity in BioFINDER-2 (n=316) and TRIAD (n=95). This metric is shown for each biomarker with screening strategies varying at sensitivity values (90%, 95%), with confidence intervals derived from 100 bootstrap trials. The bottom panel shows the positive predictive value (PPV) for each evaluated plasma biomarker at different sensitivity thresholds (90%, 95%). PPV represents the percentage of true positive tau-PET scans that can be expected among those individuals who are selected for PET scanning via plasma biomarker screening. The vertical dashed line indicates the prevalence of tau-PET positivity among the whole SCD-MCI population in BioFINDER-2 (21.2%) and TRIAD (30.5%). While PPV's for most biomarkers cross the line, plasma pTau217 was the biomarker with the best capacity to increase cost-effectiveness for tau PET screening in memory clinics. Source data are provided as a Source Data file. PET

= positron emission tomography; Aβ = amyloid-beta; pTau = phosphorylated tau; NfL = neurofilament light; GFAP = glial fibrillary acidic protein.

Supplementary Figure 5. Tau-PET scans avoided and screening accuracy in all-cause dementia individuals in BioFINDER-2 and TRIAD.

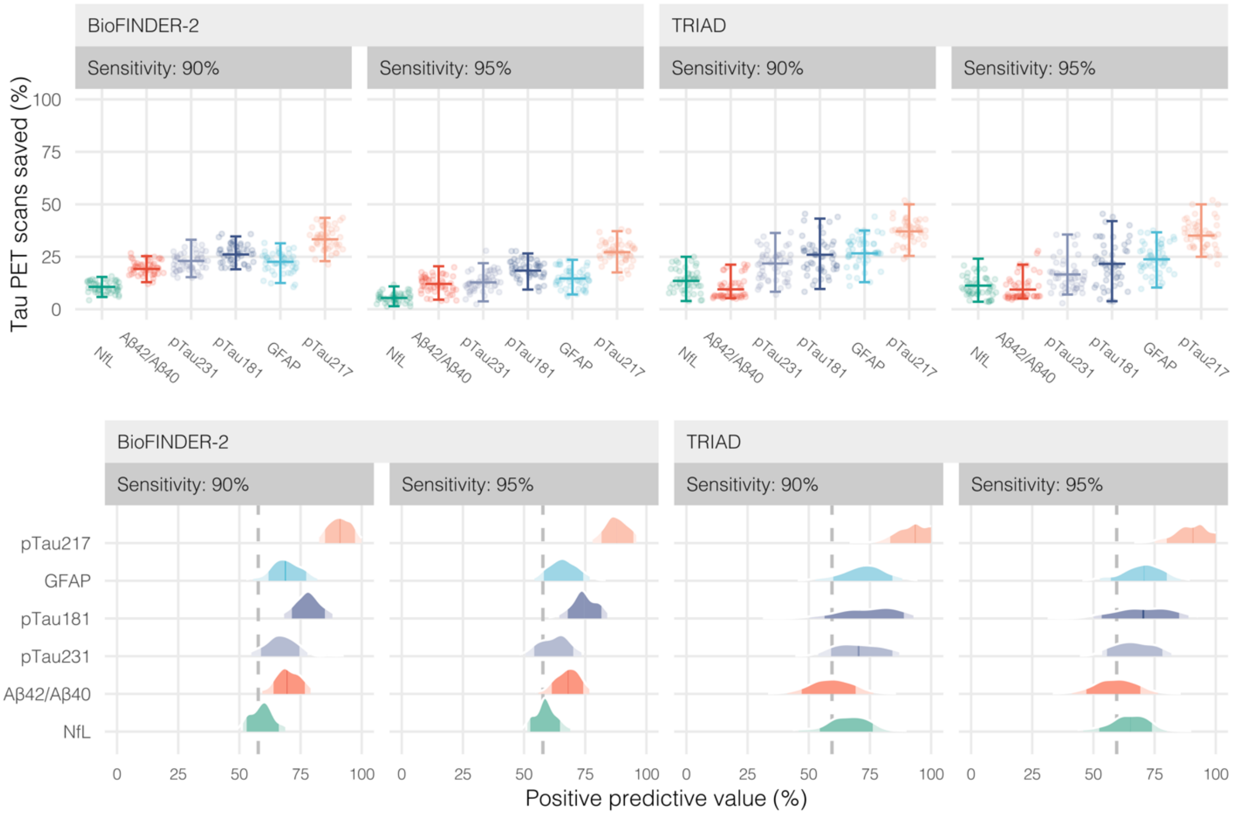

The top panels show the percentage of tau-PET scans saved when screening all-cause dementia individuals for tau-PET-positivity in BioFINDER-2 (n=232) and TRIAD (n=84). This metric is shown for each biomarker with screening strategies varying at sensitivity values (90%, 95%), with confidence intervals derived from 100 bootstrap trials. The bottom panel shows the positive predictive value (PPV) for each evaluated plasma biomarker at different sensitivity thresholds (90%, 95%). PPV represents the percentage of true positive tau-PET scans that can be expected among those individuals who are selected for PET scanning via plasma biomarker screening. The vertical dashed line indicates the prevalence of tau-PET positivity among the whole all-cause dementia population in BioFINDER-2 (57.8%) and TRIAD (59.5%). Source data are provided as a Source Data file. PET = positron emission tomography; Aβ = amyloid-beta; pTau = phosphorylated tau; NfL = neurofilament light; GFAP = glial fibrillary acidic protein.

Supplementary Figure 6. Distribution of tau-PET visual read results by plasma p-tau217 status in BioFINDER-2 participants.

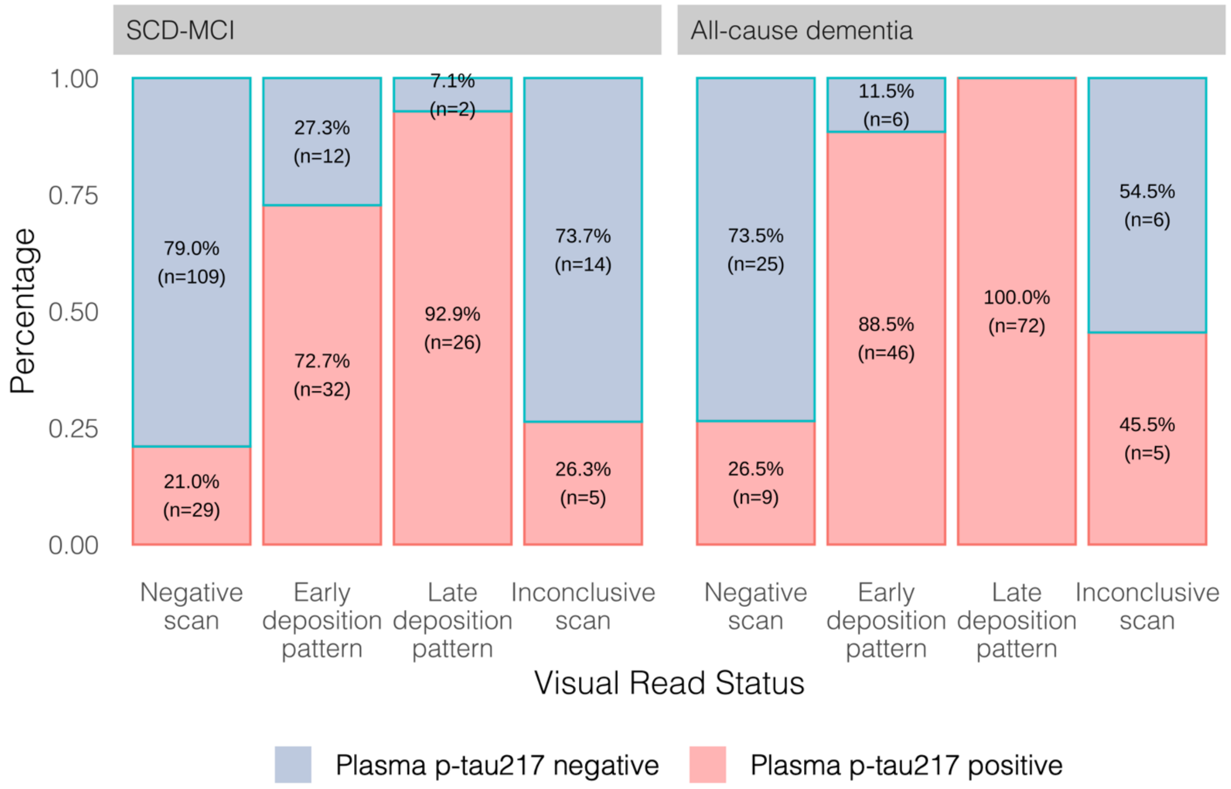

This bar plot displays the percentage of individuals within four tau-PET visual read categories, differentiated by plasma p-tau217 status using a 95% sensitivity cutoff. The x-axis categorizes the visual read outcomes, ranging from negative scan to inconclusive results. Blue bars indicate the proportion of individuals with negative plasma p-tau217, while red bars represent those with positive plasma p-tau217. The plot is divided into two panels: the left panel illustrates results for participants with Subjective Cognitive Decline-Mild Cognitive Impairment (SCD-MCI; n=312), and the right panel for patients with all-cause dementia (n=227). Source data are provided as a Source Data file. PET = positron emission tomography; pTau = phosphorylated tau.

Supplementary Figure 7. Raw cognitive trajectories of prognostic analyses based on the plasma p-tau217 referral cutoff.

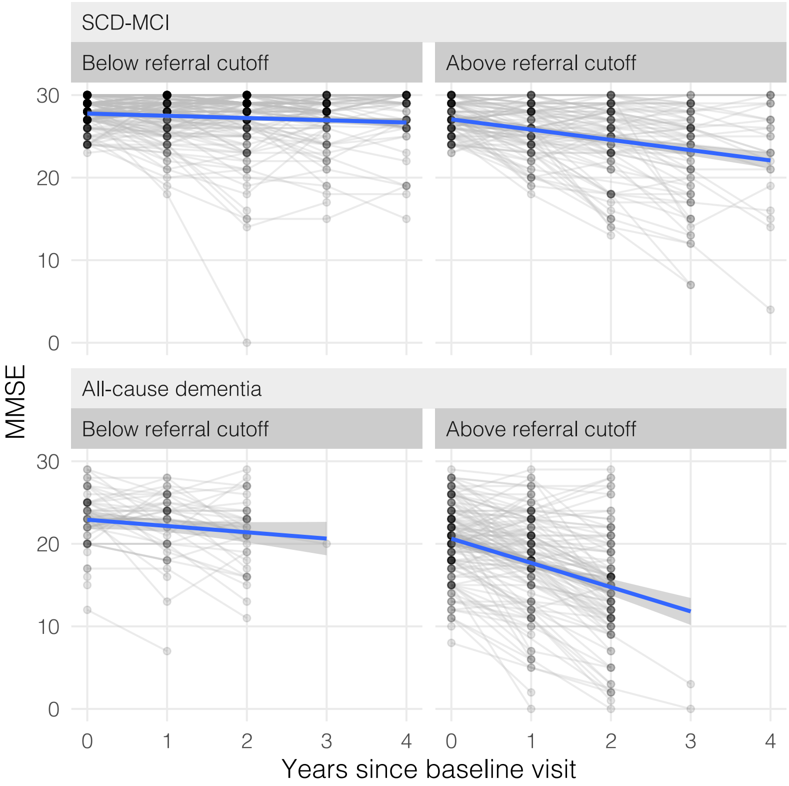

\

This plot shows the longitudinal cognitive trajectories of SCD-MCI (n=266) and all-cause dementia (n=180) participants according to the 95% sensitivity cutoff using plasma p-tau217 as a screener as measured with the Mini-Mental State Exam (MMSE). Source data are provided as a Source Data file. PET = positron emission tomography; pTau = phosphorylated tau; SCD = subjective cognitive decline; MCI = mild cognitive impairment.

Supplementary Table 1. Negative and positive predictive values for each biomarker and screening strategy in BioFINDER-2.

Biomarker	Sensitivity	Negative Predictive Value (NPV)	Positive Predictive Value (PPV)
Aβ42/Aβ40	0.9	85.1% (77.9-88.8%)	45.0% (38.5-51.5%)
	0.95	89.3% (81.2-93.6%)	42.5% (36.4-49.7%)
	0.975	93.1% (83.3-97.1%)	40.6% (33.7-47.7%)
GFAP	0.9	89.9% (85.0-93.0%)	50.0% (40.2-60.1%)
	0.95	93.0% (86.6-95.9%)	44.9% (37.4-53.7%)
	0.975	94.4% (85.7-97.5%)	40.6% (33.7-49.4%)
NfL	0.9	81.6% (66.7-88.1%)	39.8% (33.2-47.9%)
	0.95	86.4% (72.0-92.3%)	38.8% (33.1-45.8%)
	0.975	90.9% (66.7-95.6%)	37.9% (32.5-43.9%)
pTau181	0.9	88.5% (82.9-92.7%)	48.6% (39.4-58.2%)
	0.95	91.7% (84.2-95.8%)	44.5% (36.5-55.4%)
	0.975	94.7% (88.2-97.4%)	42.0% (34.4-51.1%)
pTau217	0.9	92.5% (89.9-94.0%)	73.1% (62.2-83.1%)
	0.95	95.5% (93.3-97.3%)	67.3% (52.6-77.0%)
	0.975	97.9% (96.4-98.5%)	60.1% (45.2-72.7%)
pTau231	0.9	86.0% (79.7-90.6%)	45.1% (38.2-52.6%)
	0.95	89.5% (75.0-94.3%)	41.9% (36.1-48.0%)
	0.975	92.2% (75.0-96.8%)	39.7% (33.8-47.0%)

This table shows, for each biomarker and each screening sensitivity strategy, the negative (NPV) and positive (PPV) predictive values in all included BioFINDER-2 participants (n=548). Given the high the a priori defined sensitivities, NPVs were generally very high. When looking at the PPVs, it can be nothed that only pTau217 yield reasonably metrics in comparison to this population's rate of tau-PET positivity (37%). Confidence intervals were derived with bootstrapping (n=100). PET = positron emission tomography; Aβ = amyloid-beta; pTau = phosphorylated tau; NfL = neurofilament light; GFAP = glial fibrillary acidic protein.

Supplementary Table 2. Negative and positive predictive values for each biomarker and screening strategy in TRIAD.

Biomarker	Sensitivity	Negative Predictive Value (NPV)	Positive Predictive Value (PPV)
Aβ42/Aβ40	0.9	66.7% (0.0-86.7%)	41.1% (26.5-53.9%)
	0.95	66.0% (0.0-85.7%)	40.0% (26.5-53.0%)
	0.975	68.0% (0.0-86.7%)	40.0% (26.5-53.1%)
GFAP	0.9	87.5% (77.3-92.6%)	58.6% (47.4-71.1%)
	0.95	92.3% (85.7-95.4%)	54.3% (43.0-69.5%)
	0.975	90.9% (83.3-94.7%)	52.9% (42.9-64.5%)
NfL	0.9	71.4% (33.3-85.2%)	50.0% (34.2-60.0%)
	0.95	66.7% (0.0-83.3%)	47.1% (34.1-59.3%)
	0.975	50.0% (0.0-83.3%)	47.0% (34.1-58.7%)
pTau181	0.9	87.5% (75.1-92.9%)	60.0% (42.1-75.0%)
	0.95	90.0% (57.9-94.9%)	54.4% (36.5-69.4%)
	0.975	87.5% (0.0-94.9%)	52.5% (36.5-68.4%)
pTau217	0.9	93.3% (88.9-96.2%)	86.2% (63.2-100.0%)
	0.95	96.2% (94.4-97.1%)	77.1% (60.1-94.6%)
	0.975	96.0% (94.4-97.0%)	73.1% (56.4-92.7%)
pTau231	0.9	86.7% (71.3-92.3%)	59.0% (44.5-72.1%)
	0.95	88.9% (75.0-94.9%)	51.8% (37.3-65.6%)
	0.975	84.5% (66.7-94.6%)	50.0% (36.2-62.7%)

This table shows, for each biomarker and each screening sensitivity strategy, the negative (NPV) and positive (PPV) predictive values in all included TRIAD participants (n=179). Given the high the a priori defined sensitivities, NPVs were generally very high. When looking at the PPVs, it can be nothed that only pTau217 yield reasonably metrics in comparison to this population's rate of tau-PET positivity (44%). Confidence intervals were derived with bootstrapping (n=100). PET = positron emission tomography; Aβ = amyloid-beta; pTau = phosphorylated tau; NfL = neurofilament light; GFAP = glial fibrillary acidic protein.

Supplementary Table 3. Negative and positive predictive values for tau-PET positivity defined with visual read in BioFINDER-2.

Biomarker	Sensitivity	Negative Predictive Value (NPV)	Positive Predictive Value (PPV)
Aβ42/Aβ40	0.9	85.1% (76.5-89.7%)	44.7% (38.3-51.1%)
	0.95	88.9% (82.4-93.3%)	43.1% (36.0-49.7%)
	0.975	93.3% (75.0-97.2%)	41.5% (34.1-47.5%)
GFAP	0.9	90.8% (87.2-93.6%)	51.2% (42.1-61.5%)
	0.95	93.6% (85.9-96.4%)	45.5% (37.6-54.8%)
	0.975	95.7% (84.5-97.6%)	41.9% (34.5-51.8%)
NfL	0.9	81.5% (69.3-88.8%)	40.9% (33.9-47.0%)
	0.95	86.0% (63.0-92.7%)	39.3% (32.3-46.7%)
	0.975	91.7% (66.7-95.8%)	38.5% (32.2-45.6%)
pTau181	0.9	88.6% (83.8-92.0%)	48.5% (39.6-60.0%)
	0.95	92.1% (84.9-95.6%)	44.8% (37.1-53.7%)
	0.975	94.7% (87.5-97.0%)	42.1% (34.6-48.9%)
pTau217	0.9	92.5% (89.9-94.3%)	74.0% (63.5-81.9%)
	0.95	95.9% (93.1-97.2%)	66.5% (53.9-77.0%)
	0.975	97.7% (96.5-98.4%)	57.3% (46.2-70.9%)
pTau231	0.9	86.3% (74.2-91.7%)	45.7% (37.4-52.2%)
	0.95	88.9% (75.9-94.1%)	41.6% (35.3-49.2%)
	0.975	90.9% (70.6-96.6%)	39.4% (33.4-46.2%)

This table shows, for each biomarker and each screening sensitivity strategy, the negative (NPV) and positive (PPV) predictive values in all included BioFINDER-2 participants, using a clinically validated visual read method to determine tau-PET positivity (n=539). Given the high the a priori defined sensitivities, NPVs were generally very high. When looking at the PPVs, it can be nothed that only pTau217 yield reasonably metrics in comparison to this population's rate of tau-PET positivity (46%). Confidence intervals were derived with bootstrapping (n=100). PET = positron emission tomography; Aβ = amyloid-beta; pTau = phosphorylated tau; NfL = neurofilament light; GFAP = glial fibrillary acidic protein.
