# Supplementary material for: A blood-based biomarker workflow for optimal tau-PET referral in memory clinic settings
Source: Nat Commun. 2024 Mar 14;15:2311. doi: 10.1038/s41467-024-46603-2 (PMC10940585; doi:10.1038/s41467-024-46603-2)
Supplement: Supplementary file 3 — Reporting Summary [file 41467_2024_46603_MOESM3_ESM.pdf]

nature portfolio

Corresponding author(s): Oskar Hansson, Wagner S. Brum

Last updated by author(s): 11/25/2023

Reporting Summary

Nature Portfolio wishes to improve the reproducibility of the work that we publish. This form provides structure for consistency and transparency in reporting. For further information on Nature Portfolio policies, see our Editorial Policies and the Editorial Policy Checklist.

Statistics

For all statistical analyses, confirm that the following items are present in the figure legend, table legend, main text, or Methods section.

n/a	Confirmed	
☐	☒	The exact sample size (n) for each experimental group/condition, given as a discrete number and unit of measurement
☐	☒	A statement on whether measurements were taken from distinct samples or whether the same sample was measured repeatedly
☐	☒	The statistical test(s) used AND whether they are one- or two-sided<br>*Only common tests should be described solely by name; describe more complex techniques in the Methods section.*
☐	☒	A description of all covariates tested
☐	☒	A description of any assumptions or corrections, such as tests of normality and adjustment for multiple comparisons
☐	☒	A full description of the statistical parameters including central tendency (e.g. means) or other basic estimates (e.g. regression coefficient) AND variation (e.g. standard deviation) or associated estimates of uncertainty (e.g. confidence intervals)
☐	☒	For null hypothesis testing, the test statistic (e.g. F, t, r) with confidence intervals, effect sizes, degrees of freedom and P value noted<br>*Give P values as exact values whenever suitable.*
☒	☐	For Bayesian analysis, information on the choice of priors and Markov chain Monte Carlo settings
☒	☐	For hierarchical and complex designs, identification of the appropriate level for tests and full reporting of outcomes
☒	☐	Estimates of effect sizes (e.g. Cohen's d, Pearson's r), indicating how they were calculated

Our web collection on statistics for biologists contains articles on many of the points above.

Software and code

Policy information about availability of computer code

Data collection	No software was used.
Data analysis	All statistical analyses were performed in R version 4.1.1 (www.r-project.org).

For manuscripts utilizing custom algorithms or software that are central to the research but not yet described in published literature, software must be made available to editors and reviewers. We strongly encourage code deposition in a community repository (e.g. GitHub). See the Nature Portfolio guidelines for submitting code & software for further information.

Data

Policy information about availability of data

All manuscripts must include a data availability statement. This statement should provide the following information, where applicable:

- Accession codes, unique identifiers, or web links for publicly available datasets
- A description of any restrictions on data availability
- For clinical datasets or third party data, please ensure that the statement adheres to our policy

Anonymized data will be shared by request from a qualified academic investigator for the sole purpose of replicating procedures and results presented in the article and as long as data transfer is in agreement with EU legislation on the general data protection regulation and decisions by the Swedish Ethical Review Authority and Region Skåne, which should be regulated in a material transfer agreement. Arrangements for data sharing for replication of the findings in the TRIAD data set are subject to standard data-sharing agreements, and further information can be found in the study’s website (https://triad.tnl-mcgill.com/).

Human research participants

Policy information about studies involving human research participants and Sex and Gender in Research.

Reporting on sex and gender	We used the term "sex" in the manuscript, and sex was determined based on participant self-reporting. The cohorts included recruit both self-reported men and women.
Population characteristics	A total of 548 participants in the BioFINDER-2 cohort met the criteria to be included in the present analysis based on availability of at least one of the plasma biomarkers listed below and a tau-PET scan. The average age of the cohort was 71.0 ±8.6 years, with 45% of participants being female, and an overall tau-PET positivity rate of 37% (see Methods for details). Participants had subjective cognitive decline (n=135), mild cognitive impairment (n=181), or different forms of dementia (n=225) – specifically AD dementia (n=140), bvFTD (n=16), svPPA (n=3), nfvPPA (n=1), DLB (n=24), dementia with Parkinson's disease (n=7), vascular dementia (n=16) and unclassified dementia (n=24). Among TRIAD participants meeting the inclusion criteria (n=179), patients had a mean age of 70.0±7.7 years and 57% were female, with a tau-PET positivity rate of 44%. Participants had SCD (n=23), MCI (n=72), AD dementia (n=58), and non-AD dementias (n=26) such as bvFTD (n=9), svPPA (n=1), vascular dementia (n=6).
Recruitment	BioFINDER-2 (NCT03174938) is a memory clinic based cohorts recruiting participants forwarded from primary care to secondary memory clinics in southern Sweden due to cognitive complaints. BioFINDER-2 started recruitment in May 2017. TRIAD is a cohort enrolling patients mostly from memory clinic settings, as well as voluntaries.
Ethics oversight	All patients gave their written informed consent to participate in the study. The studies were approved by the regional ethics committee in Lund, Sweden. Data were collected according to the Declaration of Helsinki. TRIAD was approved by the Montreal Neurological Institute PET working committee and the Douglas Mental Health University Institute Research Ethics Board, and written informed consent obtained from all participants.

Note that full information on the approval of the study protocol must also be provided in the manuscript.

Field-specific reporting

Please select the one below that is the best fit for your research. If you are not sure, read the appropriate sections before making your selection.

☒ Life sciences ☐ Behavioural & social sciences ☐ Ecological, evolutionary & environmental sciences

For a reference copy of the document with all sections, see nature.com/documents/nr-reporting-summary-flat.pdf

Life sciences study design

All studies must disclose on these points even when the disclosure is negative.

Sample size	We included a total of 727 individuals extensively phenotyped with clinical, imaging and biofluid-based biomarkers. There is no indication that we were insufficiently powered for these analyses, considering they evaluate the potential predictive utility of these algorithms and are not meant for ready clinical use.
Data exclusions	The main inclusion criteria was that participants presented cognitive impairment or subjective cognitive impairment and had available data for the blood biomarkers used in our analyses, as well as having available tau-PET scan results. Within the eligible participants from BioFINDER-2 and TRIAD, no datapoint or individual was excluded from any analyses.
Replication	The findings were initially established in the BioFINDER-2 cohort, and data was analyzed by WSB and NCC. Replication was successful in the TRIAD cohort, where JT analyzed the data.
Randomization	No allocation into experimental groups was performed. Thus, randomization is not relevant to this study.
Blinding	All plasma biomarker and PET analyses were performed by individuals who were blinded to the clinical data. Authors who performed the data pre-processing were blinded to demographic and clinical characteristics of individuals.

Reporting for specific materials, systems and methods

We require information from authors about some types of materials, experimental systems and methods used in many studies. Here, indicate whether each material, system or method listed is relevant to your study. If you are not sure if a list item applies to your research, read the appropriate section before selecting a response.

Materials & experimental systems

n/a	Involved in the study
☐	☒ Antibodies
☒	☐ Eukaryotic cell lines
☒	☐ Palaeontology and archaeology
☒	☐ Animals and other organisms
☐	☒ Clinical data
☒	☐ Dual use research of concern

Methods

n/a	Involved in the study
☒	☐ ChIP-seq
☒	☐ Flow cytometry
☒	☐ MRI-based neuroimaging

Antibodies

Antibodies used	Plasma phosphorylated tau 217 (p-tau217) and p-tau181 was quantified in BioFINDER-2 with immunoassays developed by Lilly Research Laboratories, and analyses were performed with the same batch of reagents. For p-tau217, biotinylated-IBA493 was used as a capture antibody and SULFO-TAG-4G102 as the detector. For p-tau181, AT270 was used as a capture antibody and SULFO-TAG-4G102 as the detector. In TRIAD, plasma p-tau217 was quantified with an immunoassay developed by Janssen R&D, using the PT3 antibody as capture and HT43 as detector. In TRIAD, p-tau231 was measured with an immunoassay developed in-house by the University of Gothenburg, using Tau12 as detector and AT270 as capture. In both cohorts, p-tau231 was measured with an immunoassay developed in-house by the University of Gothenburg, using Tau12 as detector and ADx253 as capture.
Validation	The plasma p-tau217 immunoassays here used has been previously validated and described in full detail by Palmqvist et al. (JAMA. 2020:324(8):772-781) and by Groot et al (Alz Res Ther; 2022 May 14;14(1):67). The assay version of p-tau181 used in TRIAD has been previously validated by Karikari et al (Lancet Neurol; 2020 May;19(5):422-433). The assay version of p-tau181 used in BioFINDER-2 has been previously validated by Janelidze et al (Nat Med; 2020 Mar;26(3):379-386). The plasma p-tau231 assay used in both cohorts has been previously validated by Ashton et al (Acta Neuropathol; 2021 May;141(5):709-724).

Clinical data

Policy information about clinical studies

All manuscripts should comply with the ICMJEguidelines for publication of clinical research and a completedCONSORT checklist must be included with all submissions.

Clinical trial registration	BioFINDER-2: NCT03174938.
Study protocol	BioFINDER-2: https://clinicaltrials.gov/ct2/show/NCT03174938.
Data collection	BioFINDER-2 started recruitment in May 2017.
Outcomes	The primary outcome of the study was the number of tau-PET scans avoided by biomarker screening, as well as the positive predictive value achieved by the proposed screening workflow.
